# Migration challenges among Zimbabwean refugees before, during and post arrival in South Africa

**DOI:** 10.5249/jivr.v5i1.185

**Published:** 2013-01

**Authors:** Erhabor Sunday Idemudia, John K. Williams, Gail E. Wyatt

**Affiliations:** ^*a*^North West University (MC), Department of Psychology, Mmbatho, South Africa.; ^*b*^University of California, Los Angeles (UCLA), Semel Institute for Neuroscience & Human Behavior, Department of Psychiatry & Biobehavioral Sciences, Los Angeles, US.

**Keywords:** Immigration, Trauma experience, Zimbabwean Refugees, South Africa

## Abstract

**Background:**

Zimbabweans are immigrating to South Africa with a commonly cited reason being economic opportunities. Prospects of finding employment may be a significant reason to leave behind family, friends, and community, sources that buffer and offer social support against life’s challenges. Currently, there is a dearth of research examining the motivators for Zimbabweans immigrating and the experiences encountered along the way and after arrival in South Africa. Such research is essential as large numbers of Zimbabweans may be at risk for emotional and physical trauma during this process.

**Methods:**

Two gender specific focus group discussions, each lasting 90-minutes and consisting of homeless Zimbabwean refugees, were conducted in the Limpopo Province of South Africa. A semi-structured interview assessed for experiences in and reasons for leaving Zimbabwe, as well as experiences en-route and within South Africa. Discussions were audio-recorded, transcribed, and analyzed using consensual qualitative research and a constant comparison qualitative method.

**Results:**

Three temporal themes were identified and included challenges and trauma experienced in Zimbabwe (pre-migration), during the immigration journey (mid-migration), and upon arrival in South Africa (post-migration). While there were some experiential differences, Zimbabwean men and women shared numerous traumatic commonalities. In addition to the themes, three subthemes contributing to reasons for leaving Zimbabwe, two subthemes of negative and traumatic experiences incurred mid-migration, and two post-migration subthemes of challenges were identified.

**Conclusions:**

Despite the difficulties encountered in their homeland, newly arrived Zimbabweans in South Africa may be exchanging old struggles for a new array of foreign and traumatic challenges. Reasons to immigrate and the psychological and physical toll of migration exacted at the individual and community levels are discussed. Recommendations advocating for culturally congruent mental health research, the training of culturally competent researchers and clinicians, and the development of policies that could influence the quality of life of Zimbabwean refugees are provided.

## Introduction

Over the past decade, there has been increasing attention placed upon the issue of Zimbabweans attempting to leave their country for other countries, particularly South Africa, by the news media.^1^ It has been estimated that 3.4 million Zimbabweans, which represent a quarter of the country’s population, have left Zimbabwe, making this country one of the largest contributors of immigrants in Southern Africa.^2^ Studies of international migration have cited motivators for relocation to foreign countries as economic challenges or inequalities.^3^ Supporting these studies was the significant and unprecedented numbers of Zimbabweans that immigrated to South Africa starting in 2000 with the country’s economic collapse.^1,4^ In addition, the former South African Minister of Home Affairs, Chief Inkosi Mangosuthu Buthelezi, cited three reasons for the increase of Zimbabweans. He stated that South Africa, compared to other African countries, was economically advanced, that South African employers were more willing to hire foreigners including vulnerable and undocumented (i.e., illegal) immigrants, and that Southern African countries were historically and economically connected to South Africa which, in and of itself, influenced migration patterns.^4-7^

Individual economic challenges such as poverty, unemployment, and homelessness, may not however, be the only factors contributing to international relocation.^3^ Institutional and structural factors such as civil unrest, wars, and political instability may also serve as motivators of immigration to other countries^3,8^ and have yielded almost 15 million refugees and 22 million internally displaced persons throughout the world.^9,10^ Furthermore, the consequences of civil unrest and political violence commonly result in individuals being deprived of basic necessities such as food and water^11^ and being exposed to severe and threatening experiences such as torture^12^ and/or imprisonment.^13^

The research conducted thus far cites diverse reasons as to why Zimbabweans may immigrate to other countries,^14^ with many of them having both direct and indirect influences on health. Unfortunately, empirical research focusing on the physical and mental health of immigrants in general, and on Zimbabweans specifically, is lacking and fraught with conflicting findings.^15^ Early research has suggested that immigrants were susceptible to significant mental health challenges due to the stressors encountered upon arrival in the new country.^16-22^ For example, having limited social and emotional support or experiencing difficulties in finding employment were thought to be reasons for immigrants to be at risk for mental health problems.^15,23^ Some researchers have also suggested that nativity plays an important role in health. In the National Health Interview Survey (NHIS), Asian immigrants in the United States initially had significant health advantages compared to their counterparts born in the United States.^24^ However, over time, these advantages decreased.^25^ Further, more recent research supports that immigration-related factors and mental health disorders may be different based upon gender.^26^ Thus, the issue of immigration and health is much more complicated than what earlier research suggested.

A significant limitation of the existing research is that it predominantly focuses on psychological health following immigration.^27-29^ Unfortunately, little research has addressed pre- and mid-migration experiences or focused on individual premorbid level of functioning prior to immigration which may contribute to poor health and mental health outcomes subsequent to immigration. Importantly, much of the immigration literature neglects the heterogeneity that exists within immigration groups.^23^ For example, it has only been recently that stratification of Asians into groups based on national origin has occurred.^26,30,31^ More typically, Asian immigrants are collapsed into one broad group and studied as “Asian immigrants” despite being extremely diverse in language and culture. In addition to these limitations, while the literature examining the relationship between immigration and health is increasing, very little is known in regard to Zimbabwean immigrants or more specifically, about their mental health needs.

Understanding pre-migration circumstances and the motivations for immigrating is necessary in order better address the health needs of immigrants. Such research would contribute to the identification of social determinants of health outcomes. Reports of xenophobic views in South Africa and feelings of resentment toward Zimbabweans who compete with South Africans for employment, despite being prevalent, has not deterred Zimbabweans from moving to South Africa.^32^ Violence has steadily increased over the past few years with incidents of South Africans threatening, harming, and killing Zimbabweans as strategies to persuade them to leave South Africa being frequently documented.^33-35^ Identifying the pre-existing factors and the ecological framework of the lives of Zimbabweans who immigrate to South Africa help to contextualize the reasons for immigration. Additionally, it provides a basic description of beliefs held about opportunities to improve their quality of life and more specifically, about health and access to health care.

While researchers have suggested numerous reasons as to why Zimbabweans would leave their country to live abroad, one significant reason not often cited may be embedded within a basic human rights framework. That is, Zimbabweans are confronted with enormous challenges such as poverty, drought, famine, the lack of housing and other basic resources and it may be the consequence of living with the totality of this deprivation that is driving immigration.^14^ Research that explores what influences decisions to immigrate and experiences encountered during and post-migration are all necessary in understanding the current health status, as well as the global health needs of Zimbabwean at all stages of the immigration process.

Zimbabwean immigrants, because of their lack of basic human, institutional, and structural resources, may be more accurately described as refugees, who are moving away from particular challenges, only to encounter different challenges in their lives abroad.^14^ Language is important in the discussion of Zimbabweans who leave their country, as reference to them as immigrants versus refugees provides a more telling description of who they are as a people and their motivation for relocation. Unfortunately, operationalized terminologies and definitions used in describing migrants have not been widely agreed upon, with researchers using the terms loosely or interchangeably. According to the 1951 Convention of the United Nations^36^ and the 1967 Protocol on the Status of Refugees,^37^ a refugee is defined as, “a person who owing to well founded fear of being persecuted for reason of race, religion, nationality, membership of a particular social group or political opinion, is outside the country of his nationality and is unable or owing to such fear, is unwilling, to avail himself of the protection of that country.”

While it may be questionable as to whether Zimbabweans should be classified as refugees since they are undocumented and homeless, there is support for their refugee status when the definition is expanded. In general, refugees have unique and predominantly disadvantageous characteristics including: 1) a stressful pre-migration experience in their countries of origin, which may strongly affect their subsequent adjustment;^38^ 2) a decision to migrate that is perceived as being involuntary and largely motivated by “push” rather than “pull” factors,^3,39^ which increases their risk of psychological and social adjustment problems;^40^ 3) a lack of preparation for the cross-cultural transition including the lack of financial and tangible resources such as food, clothing, and shelter but also proficient language skills;^41^ and (4) a differing cultural background than that of the targeted country.^42^ Mollica describes the experiences of refugees as being a “social earthquake,” requiring psychological intervention and research.^43,44^ Zimbabwean immigrants in South Africa may experience all of these and thus, can be defined as refugees. As such, it is important to examine their experiences before, during, and after immigration and explore their experiential appraisal of this transition.

While there is a growing body of evidence showing that war and basic human rights violations can result in psychological distress, research tends to examine experiences as pre- or post-migration with most of the attention placed upon the latter.^45-48^ Thus far, little has been documented among Zimbabweans on the psychological appraisal and consequences of their experiences across the “lifespan” of their migration. Research that focuses specifically on Zimbabwean refugees is needed as there is evidence suggesting that they may be at excessive risk for psychiatric morbidity due to migration experiences.^47^

The purpose of this formative qualitative study was threefold. First, it attempted to examine and identify pre-migration stressors and reasons influencing Zimbabweans to immigrate to South Africa. Second, it attempted to examine and identify mid-and post-migration stressors that may impact the transition into a foreign country. Finally, it attempted to inform and provide recommendations for health care providers about the physical and mental health needs of Zimbabwean refugees.

## Methods

**Participants**

After receiving Institutional Review Board (IRB) approval for the protection of human research subjects from the University of California, Los Angeles (UCLA) and the University of Limpopo (UL), recruitment of study participants began. Recruitment involved passive distribution of study fliers posted at public locations and facilities such as Non-Governmental Organization (NGO) buildings, work centers, local health and community sites, and shopping malls in the Polokwane, Limpopo Province of South Africa. Polokwane is a popular destination for Zimbabweans entering South Africa and these locations were identified as being frequently visited by Zimbabwean refugees. 

Potential participants who expressed interest in the study were screened for eligibility. The eligibility criteria included being 18 years of age or older, male or female, English-speaking, first-entry Zimbabwean, homeless, having a minimum of a one-month period of entry into South Africa, and willing to participate. Those who were found to be eligible were invited to participate in a gender-based focus group discussion (i.e., women’s and men’s groups). Twenty eligible individuals, 10 women and 10 men, were scheduled for the women’s and men’s focus group discussions, respectively.

**Procedures**

Prior to the focus group discussions, the purpose of the study was explained to participants, as were risks and benefits. Informed consent was obtained by the principal investigator who also conducted the focus group discussions. The gender-based focus group discussions, each lasting 90-minutes and scheduled on different days, took place in a confidential and conveniently located community room in a local mall in Polokwane. The group sessions were audio-recorded and transcribed verbatim. To ensure confidentiality, no personal identifiers were included in the transcripts of the group discussions. Once informed consent was obtained, nametags were distributed, whereby participants were free to print a pseudonym. Upon completion of the focus group discussions, participants were asked to complete a short demographic survey. Participants received modest incentives for their participation in the study, which included a gift voucher of 35 rand (approximately $6 U.S.) and a transportation fare of 15 rand (approximately $2.50 U.S.). 

**Setting**

The name of the town of Polokwane means “place of safety” and was formerly known as Pietersburg between 1886 and 2002. Polokwane is the capital of Limpopo Province, South Africa and is located approximately midway between Pretoria and the Zimbabwean border. According to the 2001 census conducted by Statistics South Africa, the population of the greater Polokwane municipality was 302,957, of which 91.2% were Africans, 7.2% were white, 0.9% were Colored, and 0.7% were Asians, with approximately 10% of the population residing in the municipal area. Polokwane serves as the economic hub for the greater part of the northern region.

**Measures**

A semi-structured focus group interview guide was developed which assessed for three broad constructs: 1) motivators for leaving and experiences encountered in Zimbabwe and expectations for life in South Africa; 2) experiences encountered while immigrating to South Africa; and 3) experiences encountered since arriving in South Africa. Questions asked about both personal and witnessed experiences, as well as the appraisal of these experiences. Probes were used when necessary to solicit additional or more detailed responses. 

**Data Analysis**

Consensual qualitative research (CQR)^49,50^ and a constant-comparison method of data analysis based in grounded theory^51^ were used in the data analysis. The analysis team consisted of three members, including the principal investigator and two co-investigators. The constant comparison method requires investigators to examine and compare one piece of qualitative data (i.e., sentences, themes, etc.) to another piece of data. In the first phase of analysis, individual members read the sections of each transcript that focused on the three broadly defined constructs. The investigators then met together to discuss the phenomena they identified relevant to experiences prior to leaving Zimbabwe, while en-route to, and after arrival in South Africa. Major themes identified from the empirical data (i.e., within the transcripts) were summarized in matrix form and circulated among the investigators for feedback. In the second phase of analysis, the investigators met to discuss identified themes across transcripts and then to identify common subthemes. Themes and subthemes were then applied consistently to quotations across both transcripts (i.e., coding) using Atlas.ti software to facilitate coding and manage the data.

## Results

A total of 28 individuals were screened for the study with 23 being eligible and 5 being ineligible. Three individuals were unable to attend on the date of the scheduled group. Thus, the final sample consisted of 20 Zimbabwean refugees (10 women and 10 men). The mean age was 26.5 years (SD = 6.85) with the range being 18 to 44 years. The majority of participants had a secondary education level (85%) with 10% and 5% reporting a primary and tertiary education level, respectively. The mean length of stay after arriving in South Africa was 3.3 months. Marital status while living in Zimbabwe, as well as post arrival in South Africa, was assessed. While living in Zimbabwe, 60% reported being married and 40% reported being single. However, while living in South Africa, only 25% reported being married and 75% reported being single. Reasons for the change in marital status included the stress of leaving Zimbabwe for South Africa, and economic and political challenges that strained the relationship and family unit.

The brief survey also asked participants to select reasons for leaving Zimbabwe from a given list. Primary reasons were reported as financial hardship (65%), health problems (10%), and political problems / instability (5%). All of these reasons were reported by 20%. After arriving in South Africa, 65% remained unemployed and 50% reported having health challenges. This information was further explored in the qualitative focus groups.

Three temporal themes, consistent with the broad constructs of the semi-structured interview, were identified and included reasons for leaving and challenges encountered while living in Zimbabwe, and challenges and traumatic experiences while immigrating to and post-arrival to South Africa. While there were some experiential differences, Zimbabwean men and women shared similar traumatic commonalities. In addition to the themes, three subthemes contributing to reasons for leaving Zimbabwe, two subthemes of negative and traumatic experiences incurred while travelling, and two subthemes of challenges upon arriving in South Africa were identified.

**Reasons for Leaving Zimbabwe (Pre-Migration)**

Challenges encountered in Zimbabwe were similar for both women and men in regards to the availability of resources. However, the two groups differed in reporting political and civil unrest as being a motivator for immigrating to South Africa. Three subthemes were identified and included the following:

**1. Lack of Basic Resources and Employment.**Resources such as food, water, and shelter were identified as encountered challenges that made it extremely difficult to remain in Zimbabwe. As well, the lack of employment made it difficult to purchase what resources were available.

Water is so much a problem. We have to fetch water almost three, four, five kilometers from where we stay…At Zimbabwe there’s just no job, we got no money. [FG-Female A]

I came here because I was running away from hunger and my husband was not employed there. The company was closed and he came here and looked for work. I followed my husband. [FG-Female B]

It’s like when we left that side…if only you could go to work and they pay you, and you go to the shop, but there is nothing you can buy because what is there, is too expensive. No jobs. [FG-Male A] 

**2. Lack of Health Care and Medication.**Heath care was reported to be a significant challenge due to the cost. Further, if individuals could afford to see a health care provider, there was the additional challenge of being able to afford the medication for treatment. Participants largely believed that health care, including the availability of medication, would be easily accessible in South Africa.

It’s [health care] not easy. Even now it’s not easy. So health-wise in Zimbabwe…you heard about it? There is cholera, no water, no electricity. There is nothing you can do. [FG-Female B]

It’s [heath care] too expensive and there’s also the problem of my medication. Medication is very expensive. So here in South Africa I’m treated for free. [FG-Female C]

**3. Political and Civil Unrest and Violence. **Unlike the women, several men reported political reasons and violence as a motivator to leave Zimbabwe.

Those guys from that side, they are going to force you to do something that you do not like…if you say you are not a politician, they are going to force you to join a party…So when you are in that situation, you are going to force yourself to run away or be a refugee. So most of the guys do not have papers, some of them they have got papers, but we differ… Some go to South Africa and have their passports…they just say they want to go to that side (South Africa) for a better life. [FG-Male B]

I’m someone elderly. I worked for the Zimbabwean government for twenty years as a civil servant, a senior civil servant for that matter. Unfortunately, the political situation was rising to an extent where we could not stand and I was fired because of that. When I was fired, I worked for private companies, even non-governmental organizations. But unfortunately, they were following me to the extent that I saw that my life was in danger. So I forced myself into this country without the relevant papers. My passport was stolen when I was going to Botswana sometime before but I had to force myself to come here because the situation was bad. My town, my whole area, even my kids were beaten because of me. [FG-Male C]

**Challenges and Experiences En-route to South Africa (Mid-Migration)**

The discussion of challenges encountered while travelling to South Africa yielded one subtheme experienced by both women and men and one subtheme that was uniquely reported by women. Regardless of gender, Zimbabwean refugees reported witnessing and experiencing threatening and/or physical violence. The subtheme, engaging in survival sex for resources such as food and water and the exchange of being guided through “the bush,” was reported by women.

**1. Witnessing and Experiencing Threatening and/or Physical Violence.** Many participants reported witnessing other Zimbabweans being assaulted, raped, and killed. Feelings of helplessness were commonly reported with many stating that their only goal was “to keep moving and to survive.” Additionally, many shared personal experiences of trauma.

We were crossing like twenty-seven…we were twenty-seven and there were some guys helping us to cross. We gave them money so that they [would] help us cross through Limpopo bush to Beitbridge to Messina. We found some lady lying. We don’t know whether she was dead or what. But the guy said, “No! Don’t move near! Let’s just go where we are going because this place is dangerous.”… You don’t know what is going to happen. After they check around, they’ll tell you, “let’s go, let’s go, move.” Moving very fast. My experience was sad. It was so sad. [FG-Female D]

Yeah, most people died and you know sometimes, these guys took the money from the ladies they find on the way. They take away your money…You are robbed on the road. They take your clothes and leave you naked…Yes. Even phones and nice clothes, they take it. You’d find somebody crying…saying “I’ve been raped, I don’t have money”. [FG-Female E]

We didn’t use the border side. We used the forest, the guys staying in the bush, and they robbed them - their money, cell phones and women are raped; others are killed. The people there harass people in the forest. They just shoot you. They take you as if they want to assist you to cross the border. But when you are on the way they just turn their hearts and they take your money…everything you have and they’ll just leave you in the forest and you won’t know where you are going. [FG-Male D]

**2. Engaging in Survival Sex. **In addition to being sexually assaulted and raped, female participants reported that it was common for women to find themselves in situations where survival required exchanging sex for resources and services.

I remember last week it was on the newspaper, on the front page, it was saying “Zimbabwean girls having sex for 2 rands, yeah”…Because sometimes you will be so desperate…you can’t tell a Zimbabwean to give you anything…everyone don’t have. There’s a situation whereby you don’t have anything and…they sleep with those guys for a plate of rice. [FG-Female E]

**Challenges Since Arrival in South Africa (Post-Migration) **

Consistent among both women and men were feelings of disappointment and little difference existing between Zimbabwe and South Africa in regard to economic opportunities. The two subthemes that were identified included minimal opportunities to obtain resources and employment and experiences of exploitation and coercion.

**1. Minimal Opportunities to Obtain Resources.** Regardless of gender, while in South Africa, participants reported similar challenges in obtaining the same basic necessities such as food, water, and housing that were unavailable to them while living in Zimbabwe.

The other problem we face right now in South Africa is accommodation…the treatment we are facing with the people we are staying with, belittling us… I don’t know how to explain it. I don’t know how they look at us…We pay 10 rands per day where we stay…well that’s the way they charge you. And you don’t use their water. You fetch your own water. And if you don’t have [10 rands] you go and sleep outside, and you don’t feel safe…You find Zimbabweans sleeping outside there by big bite. [FG-Female F]

We only pray that we find a job so we can look for a house. Now if you don’t work like now, you go everyday and they tell you, “no job.” Almost two weeks you don’t find a job because we are so many and you see what happens whenever you stop your car there? Everyone asks for a job. So it’s hard to find a job… okay… like every Tuesday and Wednesday we go to the Anglican Church on Devenish Street. They give us bread and soup. Half bread or a loaf. If we are many, they give you one only…And sometimes if you are lucky, some people have found some jobs. [FG-Female G]

Things are tough here. We came here expecting that we would get better opportunities in life. But the situation here is almost like the situation back home. There’s no employment here. When you don’t have employment then you have a situation whereby you got difficulties; whereby there’s no accommodation, food and basic things…everything will be troubling you because there’s no employment. And here you don’t even know when we are getting jobs, you see? So that’s the difficulties we are facing in South Africa. [FG-Male E]

The problem we are facing here in South Africa is accommodation. Jobs as well. Food as well. We expected greener pastures here, but it’s not the way we were expecting. At other times you think of back home but you keep on fighting and maybe things will be alright as time goes on. But life is hard here. [FG-Male F]

**2. Experiences of Exploitation and Coercion. **Many participants shared how vulnerable they were due to their immigration status. That is, if they were fortunate enough to get employment, there were no guarantees that they would receive appropriate wages or get compensated at all despite having worked. Also, many participants shared situations of being exploited and/or coerced.

Some kilometers away, we went to a farm, we worked for almost two or three hours on the farm and we agreed that he is going to give us sixty rands. So as we were almost a little bit finishing, he started being curious… and he chased us, he chased us with his dogs. We were almost bitten by the dogs. It was four of us… And he didn’t give us our money…We didn’t even have money for transport. We had to run and wait by the roadside there. [FG-Female H]

Then on the river, there were problems because helicopters were moving up and down. There were South African soldiers on the other side [South Africa] and we had to pay a little royalties to them so that they released us…There is always someone you have to pay…But even if you are crossing the border, you are supposed to pay them even if you’ve got your passport with you… If you don’t have anything, they say go back. So even if you’ve got the papers, it’s going to be a problem. [FG-Male G]

## Discussion

While all of the participants had some awareness of the challenges that the journey to South Africa would entail, the hopeful expectations of the Zimbabwean refugees participating in this qualitative study were not met post arrival in South Africa. These Zimbabweans left for South Africa expecting to find an abundance of opportunities and “a land flowing with milk and honey.” The basic and prevalent premise was that the risks in immigrating to South Africa were worth taking compared to living with deprivation and for some, physical harm. The fear of violence was more commonly reported by the Zimbabwean men who identified the political instability of Zimbabwe as posing threats to them and their families and communities. Unfortunately, the Zimbabwean refugees reported financial challenges, health care problems, and the lack of access to basic resources that were similar to what existed in Zimbabwe. As well, they encountered hostile and xenophobic attitudes and physical endangerment. Daily apprehension and fear left many wishing for assistance in returning to Zimbabwe where at least they had family and friends and a familiar community. This acknowledgment underscores that social support may be critical to well-being despite very few of the participants specifically mentioning it. In addition to future research examining the impact of social support as a buffer against psychological distress,^23^ understanding the effects of migration on marriage and family will be important to study. This is highlighted by the finding that seven of the twelve participants who reported being married in Zimbabwe were no longer married post-migration.

The experiences that these Zimbabwean refugees encountered en-route were laden with life-threatening situations. While they were able to openly discuss these experiences, very few acknowledged the psychological impact of these experiences and most minimized the expression of any emotional distress. For example, one female participant who had witnessed another young women being raped simply stated, “But she’s now okay. She’s fine. She was raped but she is fine.” This example represents how many of the participants discussed and appraised their experiences. That is, they acknowledged having very little control over their environment and therefore, the main objective was to physically survive their experiences. If survival was possible, they felt the outcome was adequate. Thus, there was a significant focus on how experiences affected the body (i.e., physical health and staying alive). Understanding how Zimbabwean refugees express distress may need to be explored within a cultural context. Emphasis solely on psychological symptoms using western assessments may inadvertently neglect “cultural” symptoms of distress.

Only one participant acknowledged the psychological impact of the immigration process. This male participant stated, “Many Zimbabweans are stressed…they suffer.” Attempts to further discuss the psychological impact of their experiences and current living situations resulted in participants acknowledging that Zimbabwean refugees simply had to do whatever is necessary to survive. Statements such as, “what is there to do” and “you just have no choice, you do what you have to,” were frequently expressed. Despite English being one of the official languages in Zimbabwe, in-depth exploration of psychological distress may have yielded varied responses if conducted in other indigenous languages.

When specifically asked about coping strategies used to address traumatic experiences and the ongoing challenges, almost all of the participants identified ways to obtain resources (i.e., food, water, clothing, etc.) and did not mention any psychologically based strategies. For example, engagement in exchange sex (i.e., prostitution) and crime (i.e., stealing) and for the few “fortunate” ones, obtaining small jobs such as dish washing in restaurants, street cleaning, and becoming house servants or working on farms, were identified. None of the participants discussed seeking and/or utilizing psychological, social, or spiritual support. Coping was synonymous with strategies for acquiring the basic necessities of survival and not with mental health. Culturally congruent research that explores symptoms of distress and strategies for coping among Zimbabwean refugees is greatly needed. 

The Zimbabwean refugees in this small qualitative study overwhelmingly shared similar experiences that could be temporally framed into pre-migration, mid-migration, and post-migration. Many of the challenging socio-cultural, structural, and institutional factors that they experienced, such as lacking housing (i.e., having an unpredictable and largely homeless life), having vulnerability factors to being exploited and/or coerced (i.e., being poor or an undocumented immigrant), and having a history of witnessing and/or experiencing traumatic events, were seen across all the migration stages. These highly prevalent factors have been previously reported as being associated with and predisposing for poor mental health outcomes.^47,52^ Also, research suggests that immediate past trauma, lack of functional social support, and concurrent stressful life events are all correlated with greater perceived threat or danger, harassment from the police and immigration officers, and psychological distress including feelings of shame.^53-55^ Thus, there is significant evidence underscoring the need for research that examines the psychological health needs of Zimbabwean refugees prior to, during, and after immigration.

Many questions remain concerning trauma experiences and migration. Research needs to examine whether multiple and cumulative traumatic experiences place individuals at greater risk for worse health outcomes than a single traumatic event. This will be particularly important for Zimbabwean refugees who report having a large stress burden prior to immigration. Another issue to explore is whether the temporal experience of the trauma (i.e., age of occurrence) impacts health outcomes and whether it makes a difference if it occurs pre-, mid- or post-migration. The effects of immigration for individuals at diverse developmental stages (i.e., children and adolescents), both with and without trauma is largely lacking. Only recently has research started to examine the impact on the psychological health of children who have experienced immigration.^56^For these Zimbabweans refugees, obtaining basic necessities was the priority, while mental health issues were minimized or perhaps, to be dealt with at another time.

There has been some research that examined the impact of immigration and has found that there are both positively and negatively associated health outcomes.^57,58^ Unfortunately, little research exists on the unique challenges that Zimbabweans face in their country, as well as those encountered while immigrating. Understanding the impact of the socio-cultural, institutional, and structural stressors on both their physical and psychological health has been minimally studied. Furthermore, little research has been conducted to examine the psychological stressors and mental health outcomes such as posttraumatic stress disorder or depression mid-and post-migration. While this study attempted to document some of these challenges, it did have limitations. Due to being a small preliminary study with limited resources, it included only two focus groups with a total of twenty participants and thus, findings are not generalizable and may be influenced by group effects. Also, the study occurred in one specific region of South Africa and does not illustrate the diversity of the country or of the people. Finally, conducting research with refugees may be elusive and challenging due to trust issues inherent in working with this population and may therefore impact who participates. For example, this study was limited to English-speaking Zimbabweans. Nevertheless, this small qualitative study emphasizes the need for and frames further research.

This study attempted to explore the lived experiences of undocumented Zimbabwean refugees and how these experiences affected their daily lives. Their experiences clearly support the need for culturally congruent social and mental health services. The effects of trauma, regardless of whether the individual identifies and appraises the experience as negative, need to be evaluated especially among this population. Understanding the sequelae that may be associated with or a consequence of the immigration path of Zimbabwean refugees who may not express psychological distress despite experiencing stressful and traumatic events, is greatly needed. To address this, the following recommendations are suggested:

1. Culturally congruent research, using appropriate measures, should be conducted exploring the life experiences at all stages of migration for Zimbabwean refugees. This may require that investigators first develop and / or test already established measures with Zimbabwean populations to ensure that they are culturally appropriate. Attention to traditional concepts, respect for heterogeneity among Zimbabweans and an understanding of historical factors are essential. 

2. Investigators must be culturally competent (i.e., bilingual and bicultural).

3. Research must include adequate samples in order to formulate conclusions with regard to within and / or across group differences.

4. Importantly, experts in policy should be included in this research as health services will need to address structural challenges at all stages of migration and ideally, should garner support from both the Zimbabwean and South African governments.

In conclusion, this research is only the initial step in understanding the toll exacted by immigration trauma. Study findings and recommendations serve to guide future research which is pivotal in the development of intervention and treatment programs offered by culturally competent health care providers. Culturally congruent research that includes larger samples, builds upon these preliminary qualitative findings and explore health policy issues are necessary in order to truly understand the health needs of newly immigrated Zimbabweans in South Africa.
